# Glycosylation influences activity, stability and immobilization of the feruloyl esterase 1a from *Myceliophthora thermophila*

**DOI:** 10.1186/s13568-019-0852-z

**Published:** 2019-08-12

**Authors:** Cyrielle Bonzom, Silvia Hüttner, Ekaterina Mirgorodskaya, Sun-Li Chong, Stefan Uthoff, Alexander Steinbüchel, Raymond M. D. Verhaert, Lisbeth Olsson

**Affiliations:** 1 Division of Industrial Biotechnology, Department of Biology and Biological Engineering, Chalmers University of Technology, 412 96 Gothenburg, Sweden; 2 Proteomics Core Facility, Sahlgrenska Academy, University of Gothenburg, 405 30 Gothenburg, Sweden; 3 Institut für Molekulare Mikrobiologie und Biotechnologie, Westfälische Wilhelms-Universität Münster, Corrensstraβe 3, 48149 Münster, Germany; 4 Department of Environmental Sciences, King Abdulaziz University, Jeddah, Saudi Arabia; 5 ProteoNic BV, J.H. Oortweg 19-21, NL-2333 CH Leiden, The Netherlands; 6 Present Address: State Key Laboratory of Subtropical Silviculture, School of Forestry and Biotechnology, Zhejiang Agriculture and Forestry University, Linan, 311300 People’s Republic of China

**Keywords:** Mass spectrometry (MS), Enzyme activity, Enzyme stability, Heterologous production, *Escherichia coli*, *Pichia pastoris*

## Abstract

Heterologous protein production is widely used in industrial biotechnology. However, using non-native production hosts can lead to enzymes with altered post-translational modifications, such as glycosylation. We have investigated how production in a non-native host affects the physicochemical properties and enzymatic activity of a feruloyl esterase from *Myceliophthora thermophila*, *Mt*Fae1a. The enzyme was produced in two microorganisms that introduce glycosylation (*M. thermophila* and *Pichia pastoris*) and in *Escherichia coli* (non-glycosylated). Mass spectrometric analysis confirmed the presence of glycosylation and revealed differences in the lengths of glycan chains between the enzymes produced in *M. thermophila* and *P. pastoris*. The melting temperature and the optimal temperature for activity of the non-glycosylated enzyme were considerably lower than those of the glycosylated enzymes. The three *Mt*Fae1a versions also exhibited differences in specific activity and specificity. The catalytic efficiency of the glycosylated enzymes were more than 10 times higher than that of the non-glycosylated one. In biotechnology, immobilization is often used to allow reusing enzyme and was investigated on mesoporous silica particles. We found the binding kinetics and immobilization yield differed between the enzyme versions. The largest differences were observed when comparing enzymes with and without glycosylation, but significant variations were also observed between the two differently glycosylated enzymes. We conclude that the biotechnological value of an enzyme can be optimized for a specific application by carefully selecting the production host.

**Electronic supplementary material:**

The online version of this article (10.1186/s13568-019-0852-z) contains supplementary material, which is available to authorized users.

## Introduction

Increasing interest is being shown by industry in utilizing enzymes for various applications, as they catalyze reactions with high specificity, and are considered environmentally friendly. To prepare sufficient quantities of enzymes for biotechnological applications, heterologous protein production is often used. Despite the considerable improvements made over the years in increasing protein production levels, production-scale and downstream processing (Zhang et al. 2017), enzymes still represent a significant cost to many biotechnological processes (Klein-Marcuschamer et al. 2012). Initially, *Escherichia coli* was used as the production host in most heterologous protein production strategies, and several variant *E. coli* strains and plasmids are currently available (Sørensen and Mortensen 2005; Kaur et al. 2018). However, *E. coli* is unable to introduce some post-translational modifications into the produced proteins. Post-translational modifications of proteins, such as glycosylation, phosphorylation, and acetylation, often take place in eukaryotic cells, and these modifications may have important physiological roles in proteins, for example regulatory, signaling or even functional ones (Mann and Jensen 2003).

There are two main types of glycosylation: *N*-glycosylation (linked to asparagine residues) and *O*-glycosylation (linked to the oxygen atom in serine or threonine residues, usually in proline-rich regions); *N*-glycosylation being the more common (Wayman et al. 2019). Glycan synthesis pathways, and therefore glycan structures, vary between mammalian, yeast, and plant cells (Nadeem et al. 2018), and may even differ within a phylum (Gusakov et al. 2008). Glycosylation may affect protein folding (Rudd et al. 1994; Hoffmann and Flörke 1998; Mitra et al. 2006; Benoit et al. 2006; Hanson et al. 2009), stability (Chu et al. 1978), aggregation (Schülke and Schmid 1988; Bosques and Imperiali 2003), substrate binding (Goettig 2016), the structural dynamics (Lee et al. 2015), and the catalytic activity (Skropeta 2009).

One of the most common eukaryotic hosts used for recombinant production is *Pichia pastoris*, a methylotrophic yeast, which is able to introduce glycosylation. Some applications using *P. pastoris* for recombinant protein production have already been brought to industrial scale (Ahmad et al. 2014). Filamentous fungi are also able to introduce glycosylation, and are often used for heterologous protein production. Among filamentous fungi, *Aspergillus* species and *Trichoderma reesei* are the dominating species for protein production (Nevalainen et al. 2005). Other filamentous fungus such as the thermophilic *Myceliophthora thermophila* (Visser et al. 2011) have caught attention. The genome of *M. thermophila* was annotated in 2011 (Berka et al. 2011), facilitating the development of an production platform (called C1) for the screening and production of enzymes (Visser et al. 2011).

**Fig. 1:**
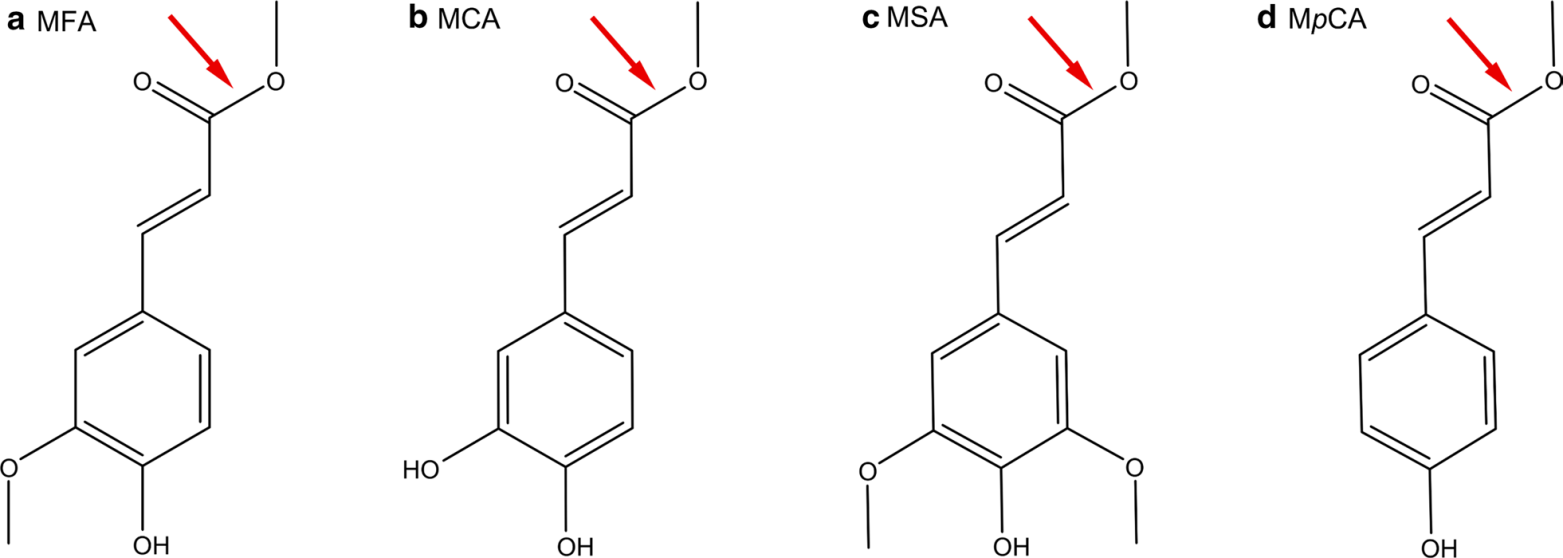
Chemical structures of the four model substrates used to assess FAE activity. The arrows indicate the bond on which FAEs act. **a** Methyl ferulate (MFA), **b** methyl caffeate (MCA), **c** methyl sinapate (MSA), and **d** methyl *p*-coumarate (M*p*CA)

Feruloyl esterases (FAEs) (E.C. 3.1.1.73) are a class of enzymes capable of hydrolyzing ester-linked ferulic acid and other hydroxycinnamic acids. In biotechnological applications aiming at utilizing plant biomass, FAEs are important enzymes that have been shown to act synergistically with other carbohydrate-active enzymes (Faulds 2010), and are therefore often present in enzymatic cocktails targeting biomass for deconstruction. The first classification of FAEs (Crepin et al. 2004) was based partly on activity on four model substrates: methyl ferulate (MFA), methyl caffeate (MCA), methyl sinapate (MSA), and methyl *p*-coumarate (M*p*CA) (Fig. 1). These hydroxycinnamic acids remain the most commonly used model substrates for FAE activity characterization.

Thermostable enzymes are highly desirable for industrial processes (Gündüz Ergün and Çalık 2016). Two major approaches have been used, and sometimes combined, to increase thermostability of enzymes: enzyme engineering and enzyme immobilization (Chapman et al. 2018; Bernal et al. 2018). Although the importance of glycosylation for protein thermostability is widely known, little information is available on how heterologous expression of glycosylated carbohydrate active enzymes affects their thermostability and/or immobilization. To elucidate the impact of glycosylation (and thus of the production host) on such enzymes, we have focused on a feruloyl esterase. *Mt*Fae1a, a feruloyl esterase from *M. thermophila* (Kühnel et al. 2012), was produced in its native host and compared to two recombinant versions of the same enzyme. The recombinant proteins were heterologously produced in *P. pastoris* (Topakas et al. 2012), which can introduce glycan chains, and in *E. coli*, which is unable to introduce glycosylation. To allow for direct comparison, we characterized the previously produced versions and the newly produced non-glycosylated one. Because of its inherent sensitivity and specificity (Halim and Anonsen 2017; Yang et al. 2017), we used mass spectrometry to identify the differences in the protein glycosylation patterns of the two glycosylated *Mt*Fae1a versions. Having confirmed both the presence and differences in glycosylation, we then investigated how this affected the physicochemical properties and activity of the enzyme. Furthermore, due to the importance of immobilization in industrial settings to protect the enzyme and allow its reuse (Datta et al. 2013; Rodrigues et al. 2013), we assessed whether glycosylation affects either immobilization into mesoporous silica (MPS) particles (Zhao et al. 1998) or the activity of the immobilized enzymes.

## Materials and methods

Chemicals were purchased from Sigma-Aldrich (St. Louis, MO, USA), unless otherwise stated.

### Cloning and production of *Mt*Fae1a versions

#### Version produced in *M. thermophila*, M-Fae

The production of *Mt*Fae1a in *M. thermophila* C1 was done as previously reported by over-production in a low background production host strain (Visser et al. 2011). The biomass-free fermentation broth was concentrated and dialyzed, and the resulting crude FAE extract was freeze-dried until use [as described for FaeB2 in (Hüttner et al. 2017)]. *M. thermophila* is also known as *Chrysosporium lucknowense* and *Thermothelomyces thermophila* (Marin-Felix et al. 2015).

#### Version produced in *P. pastoris*, P-Fae

Insertion of the *Mt*Fae1a gene in pPNic706, transformation into *Pichia pastoris* GS115 (Invitrogen) and screening of *P. pastoris* transformants were performed by ProteoNic BV. (Leiden, the Netherlands), as described for *Aspergillus niger* FaeA in (Gidijala et al. 2018). Bioreactor fermentation for the production of P-Fae was performed as described in (Gidijala et al. 2018), except that the enzyme was harvested as follows. The protein-containing culture supernatant was separated from yeast cells by continuous high-speed centrifugation, and subsequently concentrated by crossflow filtration (10 kilo Daltons (kDa) cut-off, 4 °C). The resulting protein concentrate was lyophilized, ground and stored at − 20 °C until used for further experiments. *P. pastoris* is also known as *Komagataella phaffii* (Kurtzman 2009).

#### Version produced in *E. coli*, E-Fae

In order to produce the enzyme in *E. coli*, the amino acid sequence of *Mt*Fae1a (Uniprot ID: G2QND5) was analyzed using SignalP 4.0 (Petersen et al. 2011). The first 18 amino acids were identified as a signal peptide. The signal peptide part was removed from the DNA sequence, which was then codon optimized for expression in *E. coli* and ordered as a synthetic gene from Eurofins (Luxembourg, Luxembourg) (GenBank ID: MK955161). The synthetic gene, provided in a standard cloning vector, was subcloned into a pET28 plasmid (Novagen, Merck Millipore, Burlington, Massachusetts, USA) by restriction cloning using *Nco*I and *Xho*I (Fermentas, Waltham, Massachusetts, USA) as restriction enzymes. The newly constructed plasmid, pET28-*Mt*Fae1a, encoded the same amino acid sequence as *Mt*Fae1a, minus the signal peptide, and with the addition of a C-terminal His_6_-tag. pET28-*Mt*Fae1a was transformed into an *E. coli* BL21(DE3) strain (Novagen) that had already been transformed with a plasmid coding for chaperones, pGro7 plasmid (Takara, Kusatsu, Shiga prefecture, Japan). pGro7 encodes the groES–groEL chaperones, is inducible by l-Arabinose and carries a chloramphenicol resistance marker.

Protein production was performed using 1 L of Lysogeny broth (LB) in a 2 L Erlenmeyer flask. The culture was incubated at 37 °C, under stirring at 150 rpm, until the optical density (OD_600_) reached 0.4. Chaperone production was then induced with l-arabinose (0.5 mg/L). The culture was further incubated under the same conditions until the OD_600_ reached 0.6. Protein production was then induced by the addition of isopropyl β-d-1-thiogalactopyranoside (0.2 mM), and was allowed to proceed overnight at 16 °C, 150 rpm. Cells were harvested by centrifugation (5000×*g*, 4 °C, 20 min), resuspended in 20 mL/L_culture_ of 20 mM sodium phosphate, 500 mM NaCl, pH 7.4, supplemented with DNAse I (0.05 mg/mL) and lysozyme (0.1 mg/mL). Cells were then kept frozen at − 20 °C until purification.

### Purification and analysis of the enzymes

#### Purification of E-Fae

Cells were thawed and lysed by sonication (three cycles of 3.5 min each, pulses: 1 s on/2.5 s off, amplitudes: 25%, 35% and 45%, using a Branson 450 Digital Sonifier^®^ (Branson Ultrasonics Corporation, Danbury, Connecticut, USA). The lysed cells were centrifuged (8000×*g*, 4 °C, 15 min), and solubly produced proteins were recovered in the lysis supernatant, which was filtered (0.4 µm).

E-Fae was first purified using immobilized metal affinity chromatography with a nickel column (HisTrap excel, 1 mL, GE Healthcare, Chicago, Illinois, USA) on an Äkta system (GE Healthcare). The buffers used were: A_1_: 20 mM sodium phosphate, 500 mM NaCl, pH 7.4 and B_1_: 20 mM sodium phosphate, 500 mM NaCl, 500 mM imidazole pH 7.4. The sample was loaded onto a column pre-equilibrated with buffer A_1_, washed with 2% B_1_: 98% A_1_, before a linear gradient to 100% B_1_ was applied [1 mL/min, 10 to 30 column volumes (CVs)]. Fractions (5 mL) containing the target enzyme were identified using sodium dodecyl sulfate polyacrylamide gel electrophoresis (SDS-PAGE) and/or activity assay. Enzyme-containing fractions were pooled, concentrated and imidazole was removed using a 10 kDa cut-off ultrafiltration device (Amicon Ultra15 or Amicon Ultra5, Merck-Millipore). The sample was filtered (0.4 µm) before being loaded onto the next column.

A second purification step was performed using ion-exchange chromatography (IEX) with a Q-Sepharose column (HiTrap Q XL, 1 mL, GE Healthcare) on an Äkta system. The buffers used were: A_2_: 10 mM sodium phosphate, pH 6.5, and B_2_: 10 mM sodium phosphate, 500 mM sodium chloride, pH 6.5. The sample, in buffer A_2_, was loaded onto a column pre-equilibrated with buffer A_2_, and a linear gradient to 80% B_2_: 20% A_2_ was applied (1 mL/min, for 30 CVs). Fractions (1.5 mL) containing the target enzyme, identified using SDS-PAGE and/or activity assay, were pooled and concentrated using a 10 kDa cut-off ultrafiltration device.

#### Purification of M-Fae and P-Fae

The purification protocols used for M-Fae and P-Fae were adapted from Kühnel et al. (Kühnel et al. 2012). The first purification step was performed with hydrophobic interaction chromatography using a phenyl column (HiTrap Phenyl HP, 1 mL, GE Healthcare) on an Äkta system. The buffers used were: A_3_: 10 mM sodium acetate, 1.5 M ammonium sulfate, pH 5.0, and B_3_: 10 mM sodium acetate, pH 5.0. The sample, solubilized in buffer A_3_ (M-Fae at 24 mg/mL, or P-Fae at 13 mg/mL), was loaded onto a column pre-equilibrated with buffer A_3_, and a linear gradient to 100% B_3_ was applied (1 mL/min, 60 CVs). Fractions (1.5 mL) containing the target enzyme, identified using SDS-PAGE and/or activity assay, were pooled, concentrated, and desalted using a 10 kDa cut-off ultrafiltration device. A second purification step was performed using IEX, as described above for E-Fae.

#### Protein analysis

Protein quantification was performed at 280 nm with a Nanodrop 2000 (Thermo Fisher Scientific). The following parameters were used for quantification, ε_280_: 49 640/M/cm, molecular weight (MW): 29 506 Da for M-Fae and P-Fae, and MW: 30 760 Da for E-Fae. All parameters were estimated using ExPASy ProtParam tool (Gasteiger et al. 2005).

SDS-PAGE was performed with Mini-PROTEAN^®^ system from Bio-Rad (Hercules, California, USA), Stain-Free™ Precast Gels, and Precision Plus Protein™ Unstained Standard, according to the manufacturer’s guidelines. Imaging was performed with a Gel Doc™ EZ system and Image Lab™ software (Bio-Rad). Protein molecular weights were estimated using the tools included in the Image Lab™ software. The enzymes were kept frozen (− 80 °C, 5% v/v glycerol) until use.

*N*-Glycans were removed using PNGaseF (New England Biolabs, Ipswich, Massachusetts, USA) under denaturing conditions, according to the supplier’s instructions and visualized by SDS-PAGE. Deglycosylation using non-denaturing conditions was also attempted according to the supplier’s instructions. No mobility shift was observed on SDS-PAGE after a first incubation at 37 °C for 16 h (data not shown), nor after addition of fresh PNGaseF and a second incubation at 37 °C for 4 h (data not shown).

### Mass spectrometric (MS) analysis

#### Protein digestion and NanoLC/MS analysis

Purified enzyme preparations were diluted with 50 mM triethylammonium bicarbonate (TEAB) pH 8.0 to give protein concentrations of 1 µg/µL. 20 µg of each enzyme were then digested, using Pierce™ MS grade chymotrypsin (Thermo Fisher Scientific), according to the supplier’s instructions. The digestion reactions were stopped by acidification with 10% trifluoroacetic acid, and the samples were desalted using Pierce™ Peptide Desalting Spin Columns (Thermo Fischer Scientific) according to the manufacturer’s guidelines. The salt-free supernatants were dried in a vacuum concentrator and reconstituted in 2% acetonitrile in 0.1% formic acid for liquid chromatography/mass spectrometry (LC/MS) analysis.

Digested samples were analyzed on a QExactive HF mass spectrometer interfaced with an Easy-nLC1200 liquid chromatography system (Thermo Fisher Scientific). Peptides were trapped on an Acclaim Pepmap 100 C18 trap column (100 μm × 2 cm, particle size 5 μm, Thermo Fischer Scientific) and separated on an in-house packed analytical column (75 μm × 300 mm, particle size 3 μm, Reprosil-Pur C18, Dr. Maisch GmbH, Ammerbuch, Germany) using a 75 min gradient from 5.6% acetonitrile in 0.2% formic acid to 40% acetonitrile in 0.2% formic acid, followed by a step increase to 80% acetonitrile in 0.2% formic acid for 5 min (flow rate: 300 nL/min). The instrument was operated in data-dependent mode, where the precursor ion mass spectra were acquired at a resolution of 120,000 m/z range 600–2000. The 10 most intense ions, with charge states 2 to 5, were selected for fragmentation using higher-energy collisional dissociation at collision energy settings of 28. The isolation window was set to 3 m/z, and the dynamic exclusion to 20 s. MS/MS spectra were recorded at a resolution of 30,000.

#### Database search and glycosylation data analysis

Proteome Discoverer version 2.2 (Thermo Fisher Scientific) was used together with the Byonic search engine (Protein Metrics, Cupertino, California, USA) to identify glycopeptides in both FAE preparations. The precursor and fragment ion tolerance were set to 5 ppm and 20 ppm, respectively. Chymotryptic peptides cleaved after Phe, Trp, Tyr or Leu, with up to 4 missed cleavages, were accepted. The Swissprot database (taxonomy: fungi) was used as protein database. A project-specific glycan database was created to include extended high-mannose structures (up to 30 hexose residues) as well as phosphorylated mannose structures. The modified high-mannose glycan database together with methionine oxidation were allowed as variable modifications. Cysteine carbamidomethylation was set as a static modification. Glycopeptide identification was manually evaluated prior to the final assignment of the observed glycosylation forms for each glycopeptide.

The extracted ion chromatogram (EIC) peak intensities were used to determine the site-specific glycoform distribution (microheterogeneity). The EIC peak intensities of all glycopeptides, for each chymotryptic peptide were used to calculate the relative abundances of its glycoforms. The final site-specific glycoform abundances were calculated as the average value of the abundances obtained for the individual chymotryptic peptides sharing the same glycosylation site. Representative MS/MS spectra are presented in Additional file 1: Figure S1. The data (including standard deviations) used to plot Fig. 3a and b are given in Additional file 1: Tables S1, S2, respectively.

### Enzymatic assays

Four model substrates (Fig. 1): MFA, MCA, MSA, and M*p*CA and their corresponding products: ferulic acid, caffeic acid, sinapic acid and *p*-coumaric acid (*p*CA), respectively, were used for activity assays (obtained from Apin Chemicals Ltd., Abingdon, UK). All reported enzymatic activities were determined using triplicate experiments, corrected using corresponding buffer blank reactions. Activity rates were determined based on the slope of the initial linear part of the curve. Enzymes were typically assayed at approximately 10 nM for M-Fae, 15 nM for P-Fae and 25 nM for E-Fae.

#### Continuous enzymatic assays

Standard continuous assays were performed as follows. Each enzyme was allowed to react with 0.25 mM M*p*CA [10 µL of a 5 mM stock solution prepared in dimethyl sulfoxide (DMSO)] in a final reaction volume of 200 µL using a 96-well microtiter plate (Sarsted, Nümbrecht, Germany), in 100 mM sodium phosphate (pH 7.0). Reaction progress was monitored by measuring the absorbance at λ = 340 nm in a plate reader (SPECTROstar Nano, BMG Labtech, Ortenberg, Germany) set at 35 °C. The product formation was determined using standard curves obtained using known mixtures of substrate and product. The velocities were obtained by expressing the quantity of product formed per unit time. The specific activities (SAs) of enzymes in solution were determined as described in the standard assay by using 0.25 mM of MFA, MCA or MSA as substrate.

The kinetic parameters *K*_*m*_ and *V*_*max*_ were obtained for the three *Mt*Fae1a versions using the standard assay and varying the initial concentration of M*p*CA between 5 µM and 1 mM. The results were fitted by non-linear regression using the “Enzyme kinetics” module from SigmaPlot (Systat Software Inc., San Jose, California, USA), allowing for the determination of *K*_*m*_, *k*_*cat*_ (through *V*_*max*_) and in some cases of *K*_*si*_ (Additional file 1: Figure S2).

#### Stopped enzymatic assays

Standard stopped assays were performed as follows. Each enzyme was allowed to react with 0.25 mM M*p*CA (28 µL of a 5 mM stock solution prepared in DMSO) in a final reaction volume of 700 µL in 100 mM sodium phosphate (pH 7.0). Reaction mixture without the substrate was incubated at the desired temperature in a thermomixer (Eppendorf, Hamburg, Germany) for 5 min before the reaction was started by substrate addition. Samples (60 µL) were taken every minute for 10 min, immediately quenched by adding them in 180 µL 1 M sodium carbonate, and stored on ice until the end of the run. To determine the amount of product released, 200 µL of each quenched sample was transferred to a microtiter plate, and the absorbance was read at 370 nm. Product formation was determined using standard curves from known mixtures of the substrate and. The velocities were then obtained by expressing the quantity of product formed per unit time. When working with immobilized enzyme, shaking was set to 1400 rpm on the thermomixer, and an additional centrifugation step (10,000×*g*, 1 min, 4 °C) was performed before the samples were transferred to microtiter plates. The specific activities of the immobilized enzymes were determined (after immobilization in sodium phosphate buffer, pH 6.0) at 35 °C using the standard stopped assay and changing the tested substrate to 0.25 mM MFA, MCA or MSA.

To determine the optimal temperature for activity, T_opt_, (defined as the temperature at which maximal activity was observed), the activity of the enzymes, in solution or following immobilization (sodium phosphate buffer, pH 6.0), was determined using the standard stopped assay and varying the temperature used during the reaction.

### Melting temperature (T_m_) determination

The thermal stability of the enzymes was evaluated based on the melting temperature, T_m_ (i.e., the temperature at which 50% of the protein molecules are unfolded). T_m_ value was obtained by differential scanning fluorimetry on a quantitative polymerase chain reaction apparatus (Mx3005P Q-PCR, Stratagene, San Diego, California, USA) using SYPRO Orange gel stain as the dye. Samples, 20 µL (5X SYPRO Orange, 2–10 µM enzyme, 100 mM NaCl in 100 mM sodium phosphate, pH 7.0) were added to a 96-well PCR plate (4titude, Brooks Life Sciences, Chelmsford, Massachusetts, USA), and the fluorescence intensity was recorded (1 °C/min increase from 25 to 99 °C, 3 measurements/min, λ_excitation_: 492 nm and λ_emission_: 516 nm). The relevant portion of the recorded fluorescence intensity data was fitted to a sigmoid curve, allowing the determination of T_m_ (Additional file 1: Figure S3).

### Immobilization of the enzymes

The immobilization support used in this study was Santa Barbara Amorphous type 15 (SBA-15), a type of mesoporous silica (MPS) particles, which present a tunable and ordered network of hexagonal porous structures (Zhao et al. 1998). They were synthesized and characterized as previously described (Bonzom et al. 2018), and were a kind gift from Milene Zezzi Do Valle Gomes (Chalmers University of Technology). The main properties of the particles are: pore size 10.1 nm, BET surface area 439 m^2^/g and specific pore volume 1.11 cm^3^/g.

The immobilization procedure was adapted from Thörn et al. (Thörn et al. 2011). Briefly, the support material was washed with 100 mM phosphate citrate at pH 6.0, the enzymes were diluted to a final concentration of 0.2 mg/mL in the same buffer and put into contact with the support (in a micro-centrifuge tube, 44 µL/mg_MPS_, typically using 2–10 mg MPS/reaction). Incubation was done in a thermomixer (20 °C, 1400 rpm) for 15 h. Immobilization was stopped by centrifugation (5 min, 15,000×*g*, at room temperature) and the MPS bearing the enzyme was washed 3 times with the immobilization buffer to remove unbound enzyme.

To study the immobilization kinetics, adsorption of the three *Mt*Fae1a versions was followed for 24 h in 100 mM phosphate citrate buffer at pH 5.0, 6.0, and 7.0. Immobilization was performed in triplicate experiments on 2 mg MPS. The relative percentage of enzyme bound to the support was determined at each sampling time by comparing the enzymatic activity remaining in the reaction supernatant to that of a control without MPS. The activity was determined according to the standard continuous assay method.

To determine the best immobilization pH, immobilization was performed in triplicate experiments on 2 mg of MPS using 100 mM phosphate citrate buffer at pH 5.0, 6.0, and 7.0. Based on the results obtained while studying immobilization kinetics, the contact time between enzymes and support was decreased to 15 h.

### Nucleic acid sequence of the *E. coli* codon optimized gene

atgggcgcctccttacaggaagtcaccgaattcggcgataacccgaccaacatccagatgtacatctacgttccggatcagttggataccaatcctccggtcattgtagcgttacacccatgtggcggtagtgcccagcaatggttctcaggcacgcaacttccgagctatgccgacgacaatggtttcatcctgatttatccgagcacaccccatatgagcaattgctgggatattcagaacccggatactctgactcatgggcaaggtggggatgcgctgggaattgtgtcgatggtgaactacaccctggacaaacactcaggcgattcttctcgcgtgtatgcgatgggcttcagcagtggcggcatgatgacgaaccaacttgctggctcgtacccagacgtgtttgaggctggagcggtgtattccggtgttgcgtttggttgcgcagccggtgcagaaagtgcaaccccgttttcgcccaaccagacctgtgcgcaaggactgcagaaaaccgcacaggaatggggcgattttgtacggaatgcgtatgccggatatactggccgtcgtcctcgcatgcagatctttcacggcttagaggacacactggttcgccctcagtgcgctgaagaagccctcaaacaatggagcaatgtgctgggtgtcgagctgacgcaagaagtctctggcgtaccatccccgggttggacgcagaagatctacggcgatggtacgcagttgcaagggttctttggtcaagggattggtcatcagagcaccgttaacgaacagcagctcctgcagtggtttgggctgattctcgagcaccaccaccaccaccactga.

## Results

### Different production hosts yield enzyme versions with different molecular weights

**Fig. 2:**
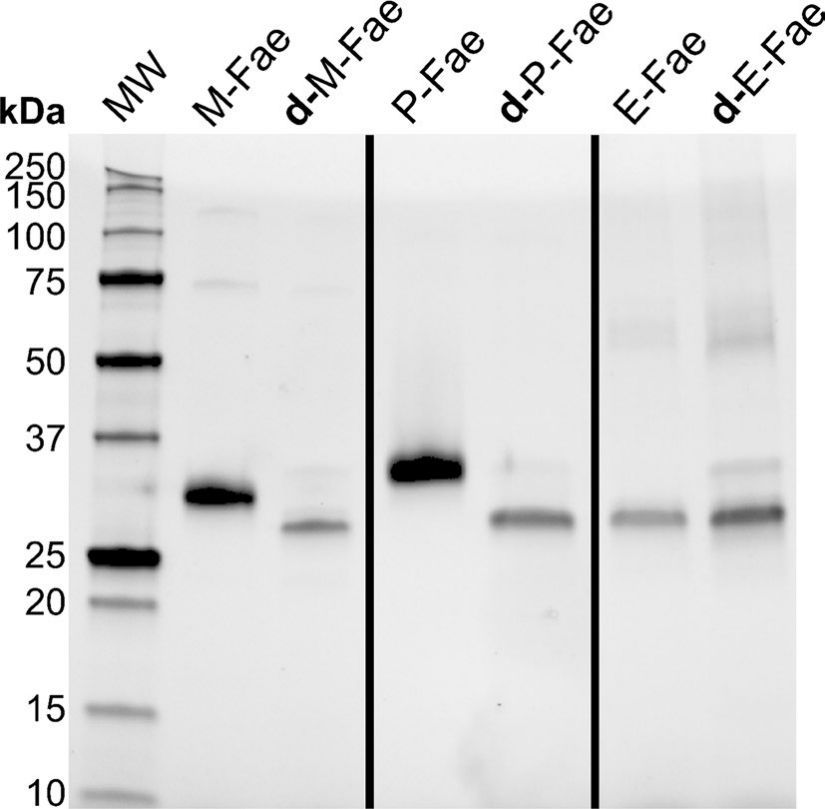
SDS-PAGE of the three purified *Mt*Fae1a versions before and after deglycosylation treatment with PNGaseF. Purified *Mt*Fae1a versions: M-Fae was produced in *Myceliophthora thermophila*, P-Fae in *Pichia pastoris* and E-Fae in *Escherichia coli*. **d**-M-Fae, **d**-P-Fae and **d**-E-Fae are the corresponding samples after deglycosylation treatment with PNGaseF. The molecular weight of PNGaseF ≈ 36 kDa. Migration of the molecular weight ladder is shown in the first lane, and corresponding molecular weights are given on the left. All three enzymes have the same amino acid sequence

The gene coding for the feruloyl esterase 1a from *Myceliophthora thermophila*, *Mt*Fae1a, was introduced into three different microorganisms: (i) its native host, *M. thermophila*, (ii) the methylotrophic yeast *Pichia pastoris*, and (iii) the bacterium *Escherichia coli*. The enzyme was successfully produced in all three host organisms, yielding the three enzyme versions: M-Fae (produced in *M. thermophila*), P-Fae (produced in *P. pastoris*) and E-Fae (produced in *E. coli*). The enzyme versions were purified to ≥ 95% (estimated by SDS-PAGE), and their apparent molecular weights determined to be 31, 33 and 28 kDa, for M-Fae, P-Fae and E-Fae, respectively (Fig. 2). The differences in electrophoretic mobility observed for the three *Mt*Fae1a versions were consistent with the prediction of two possible *N*-glycosylation sites in the amino acid sequence (Asn117 and Asn179). Their location in the protein structure, as well as the location of the Ser/His/Asp catalytic triad, can be visualized on the homology model of Topakas et al. (2012) (Additional file 1: Figure S4). After enzymatic deglycosylation of the three *Mt*Fae under denaturing conditions (Fig. 2), shifts in electrophoretic mobility (− 3 kDa for M-Fae and − 5 kDa for P-Fae) were observed. As expected, no shift was observed for E-Fae and the deglycosylated M-Fae and P-Fae migrated at the same height as E-Fae.

### Mass spectrometry analysis revealed differences in glycan chain length

**Fig. 3:**
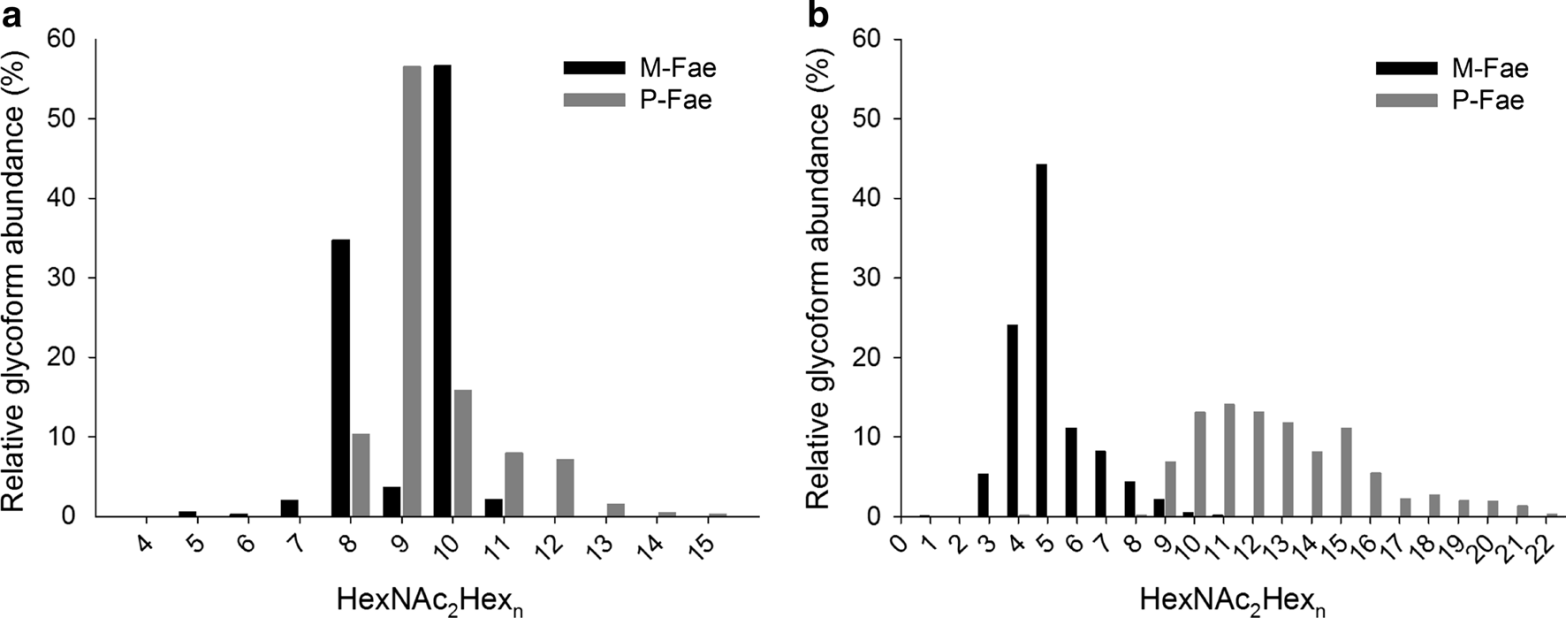
Relative glycoform abundance at the two glycosylation sites of M-Fae and P-Fae. **a** Asn 179 glycosylation site. **b** Asn117 glycosylation site. X-axis: glycoforms, HexNAc_2_Hex_n_, where “n” is the number on the axis. Hex: hexose

The two glycosylated enzymes, M-Fae and P-Fae, were analyzed using MS. In both cases, the mature (without signal peptide) *M. thermophila* feruloyl esterase *Mt*Fae1a (UniProt # G2QND5) was identified with 100% sequence coverage when *N*-glycosylation was selected as a modification (data not shown), and no *O*-glycans were detected. Both the predicted glycosylation sites, Asn117 (NYT) and Asn179 (NQT), were confirmed to be glycosylated. The observed glycoforms differed considerably between the two samples; P-Fae exhibiting overall larger glycan structures than M-Fae (Fig. 3). Furthermore, glycans containing mannose phosphate were absent from the M-Fae sample, but present at both glycosylation sites for P-Fae (Additional file 1: Table S3). No non-glycosylated peptides were observed at the Asn179 site, indicating that it was fully occupied in both enzyme versions (Additional file 1: Table S3). The Asn179 site showed the presence of similarly sized *N*-glycan structures for both protein preparations, but at different abundances for the observed glycoforms (Fig. 3a). The differences were even more marked for the Asn117 site, where *N*-glycans of different sizes were observed (Fig. 3b). M-Fae exhibited high-mannose structures from HexNAc_2_Hex_1_ to HexNAc_2_Hex_11_, while P-Fae had larger ones, extending up to HexNAc_2_Hex_22_.

### Native glycosylation confers the best biochemical properties to enzymes in solution

**Fig. 4:**
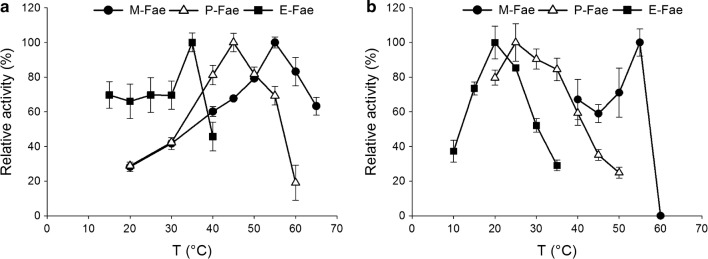
Activity of the three *Mt*Fae1a versions in solution (**a**), and immobilized (**b**), as a function of temperature. Activities determined on M*p*CA, in sodium phosphate buffer at pH 7.0. Results given are averages of three experiments, and error bars represent one standard deviation

To assess potential differences in substrate preference, the three *Mt*Fae1a versions were tested on four model compounds: MFA, MCA, MSA, and M*p*CA (Fig. 1). All three *Mt*Fae1a versions showed a preference for the least substituted aromatic substrate, M*p*CA (Table 1). Therefore, this substrate was used to determine the activity profiles of the enzymes at different temperatures and pHs (Fig. 4a and Additional file 1: Figure S5). The three enzyme versions showed significant differences in their activity profiles as a function of temperature (Fig. 4a). The optimal temperature for activity (T_opt_) was highest for the natively glycosylated M-Fae, at 55 °C, followed by the glycosylated P-Fae at 45 °C, and the non-glycosylated E-Fae at 35 °C (Fig. 4a). In contrast, the pH optimum was found to be similar for all three *Mt*Fae1a versions, in the pH range 7.0–8.0 in sodium phosphate (Additional file 1: Figure S5). The effect of the glycosylation state on the optimal pH may depend on intrinsic protein properties, as different responses have been observed in various enzymes. A fungal FAE produced in its native host *Aspergillus niger,* and in *E. coli*, exhibited similar pH profiles (Benoit et al. 2006), while the optimal pH for a tannase from *Aspergillus oryzae* differed depending on its production host (*A. oryzae* or *P. pastoris*) (Mizuno et al. 2014), and the optimal pH for a phytase from *Aspergillus fumigatus* was shifted one pH unit after deglycosylation (Guo et al. 2008).

**Table 1:** Relative and specific activities of the three MtFae1a versions on FAE model substrates

	M-Fae		P-Fae		E-Fae	
	Relative activity (%)	Specific activity (µM/min/µg)	Relative activity (%)	Specific activity (µM/min/µg)	Relative activity (%)	Specific activity (µM/min/µg)
Methyl ferulate	47.5 ± 1.3	73.7 ± 2.0	64.9 ± 0.7	95.2 ± 1.1	37.6 ± 1.6	15.7 ± 0.7
Methyl caffeate	37.8 ± 4.0	58.7 ± 6.2	38.7 ± 1.9	56.8 ± 2.8	40.8 ± 3.2	17.1 ± 1.3
Methyl sinapate	19.9 ± 0.7	30.8 ± 1.1	24.8 ± 0.9	36.4 ± 1.3	Trace^a^	Trace^a^
Methyl *p*-coumarate	100 ± 6.0	155 ± 9.3	100 ± 1.7	147 ± 2.5	100 ± 3.4	41.9 ± 1.4

Data were obtained at pH 7.0 and 37 °C, using a continuous assay. Results are presented as the average of three experiments ± one standard deviation

^a^Trace: trace activity observed (less than 0.4 µM/min/µg)

The specific activity of each *Mt*Fae1a version was determined at 37 °C and pH 7.0 (Table 1). M-Fae and P-Fae exhibited similar SAs on MCA and M*p*CA, and the highest value was observed for M-Fae on M*p*CA (155 µM_*p*CA_/min/µg_Fae_). P-Fae was, however, more active than M-Fae on the other two substrates, MFA and MSA, suggesting less stringent substrate specificity. The non-glycosylated version, E-Fae, showed a markedly reduced activity.

The kinetic parameters were determined for M*p*CA at the optimum temperature for E-Fae, 35 °C (Table 2 and Additional file 1: Figure S2). No significant differences were observed between the two glycosylated enzymes. Their apparent affinity constant, *K*_*m*_, turnover number *k*_*cat*_, and substrate inhibition constant *K*_*si*_, were within one standard deviation (Table 2). The catalytic efficiency (*k*_*cat*_*/K*_*m*_) of the non-glycosylated E-Fae was nearly 20-fold lower than those of the glycosylated enzymes. The low efficiency of E-Fae was due to both a sixfold higher *K*_*m*_ value, and a three-fold lower *k*_*cat*_ value (Table 2). These results emphasized the importance of glycosylation for *Mt*Fae1a activity. The results obtained here are consistent with those observed for a non-glycosylated FAE (Benoit et al. 2006), a recombinant tannase (Mizuno et al. 2014), and a mutated FAE (Koseki et al. 2006), for which differences in glycosylation or the lack of it, at best, had no negative influence on the overall catalytic efficiency.

**Table 2:** Kinetic parameters of the *Mt*Fae1a versions on methyl *p*-coumarate

	*K*_*m*_ (mM)	*K*_*si*_ (mM)	*k*_*cat*_ (s^−1^)	*k*_*cat*_*/K*_*m*_ (s^−1^ M^−1^)
M-Fae	0.013 ± 0.002	1.46 ± 0.29	19 ± 0.8	1.44E+06
P-Fae	0.012 ± 0.001	1.84 ± 0.28	18 ± 0.5	1.47E+06
E-Fae	0.081 ± 0.010	NA	6.1 ± 0.2	7.48E+04

Data were obtained in sodium phosphate at pH 7.0, and 35 °C. Results are presented as the average of three experiments ± one standard deviation

*NA* not applicable

### Native glycosylation contributes to pH and temperature stability

The long-term pH stability of the enzymes was evaluated at 20 °C, over the pH range 6.5–8.5 for 48 h. The measured residual activities showed M-Fae and P-Fae were relatively stable at all pHs and displayed similar behavior with residual activities above 80% after 8 h of incubation (Additional file 1:Figure S6a,b). E-Fae was much less stable, and residual activity decreased below 80% after only 1 h of incubation in some buffers (Additional file 1: Figure 6c).

The thermal stability of the enzymes was evaluated by estimating their T_m_ (Additional file 1: Figure S3). M-Fae and P-Fae exhibited higher T_m_ values than E-Fae, in agreement with the previously described thermo-stabilizing effect of protein glycosylation (Chu et al. 1978). Interestingly, despite both being glycosylated, the T_m_ value of M-Fae was found to be 8 °C higher than that of P-Fae; being 60.1 °C ± 0.2 and 51.9 °C ± 0.1, respectively. The T_m_ value of E-Fae was 42.0 °C ± 0.1.

### Glycosylation influences the immobilization process and the properties of immobilized enzymes

**Fig. 5:**
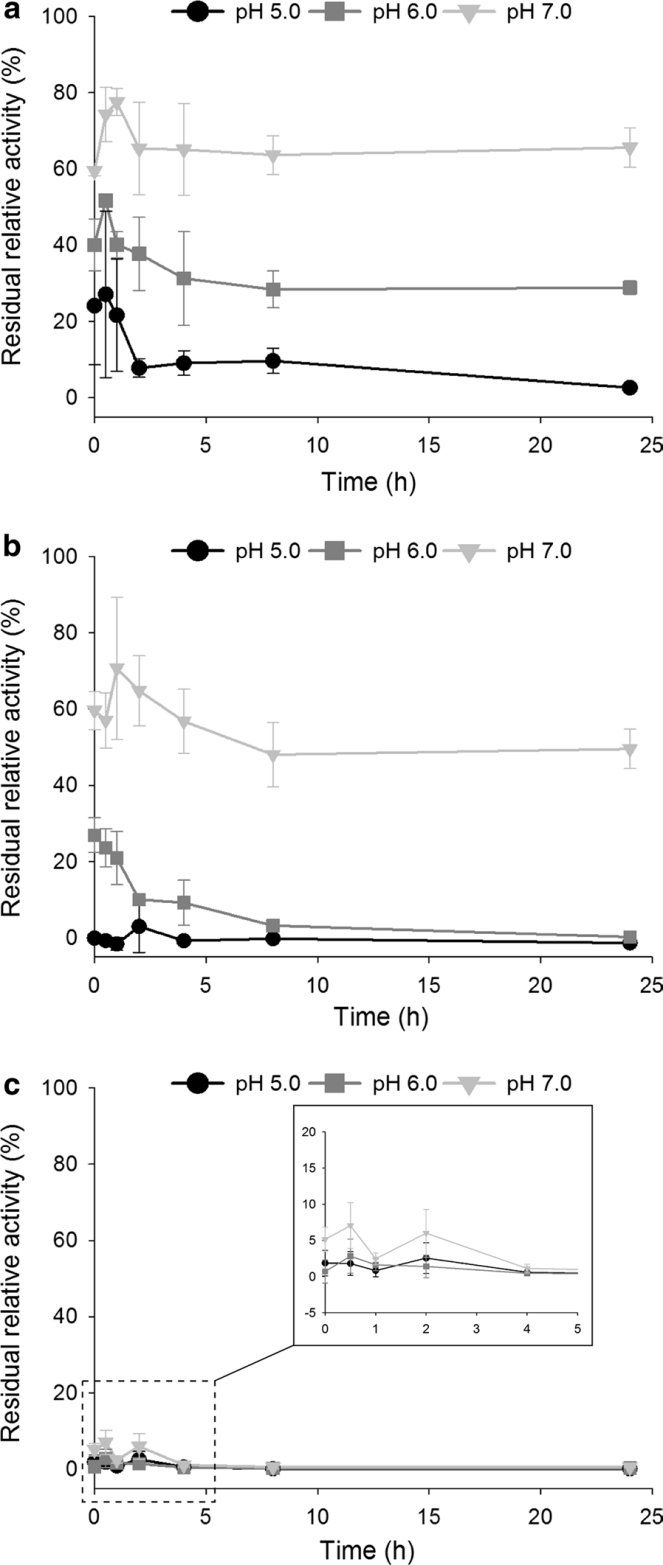
Immobilization kinetics of the three *Mt*Fae1a versions. **a** M-Fae. **b** P-Fae. **c** E-Fae, the inlay shows an enlargement of the first 5 h of adsorption. Adsorption of M-Fae, P-Fae and E-Fae in mesoporous silica particles was followed for 24 h in sodium phosphate. The results are presented as the enzymatic activity in the supernatant relative to the activity measured in a sample that had not been in contact with MPS. The values given are averages of three experiments, and error bars represent one standard deviation

The efficiency of enzyme immobilization in porous materials by adsorption depends on several factors, including the apparent surface charge of the protein and the capacity of the protein to enter the pores, both of which could be influenced (positively or negatively) by protein glycosylation. Therefore, the influence of glycosylation on the adsorption kinetics of the three enzymes was evaluated (Fig. 5). The behavior of the three enzyme versions differed, both in terms of the rate of immobilization and final yields. For the non-glycosylated E-Fae, less than 10% of the enzymatic activity was detected in the supernatant from the first sampling time (t = 0), under all conditions tested (Fig. 5c). Which indicates that over 90% of the enzyme was immediately immobilized. The glycosylated enzymes, M-Fae and P-Fae, exhibited pH-dependent immobilization kinetics, as is expected when immobilization is driven by electrostatic interactions (Fig. 5a, b). The fastest immobilization was observed at the lowest pH. After 24 h of contact, the immobilization yields ranged from 34 to 97% for M-Fae, and from 50 to 100% for P-Fae (Fig. 5a, b).

It has previously been shown that the pH at which immobilization is performed influences the subsequent enzymatic activity (regardless of the pH used during the reaction) (Thörn et al. 2013). The activity of the enzymes, immobilized at different pHs, was therefore determined (Additional file 1 Table S4). All three *Mt*Fae1a versions exhibited their highest specific activity when immobilized at pH 6.0. After immobilization, the substrate preference of the enzymes was unchanged (Table 3), but their specific activities were drastically lower than those observed for their free counterparts (Tables 1, 3). A decrease in activity upon immobilization has been observed for another enzyme immobilized in SBA-15 (Qu et al. 2014), and may be attributed to conformational changes in the enzyme upon immobilization, or limited substrate diffusion (Rodrigues et al. 2013).

**Table 3:** Relative and specific activities of the three immobilized *Mt*Fae1a versions on FAE model substrates

	M-Fae		P-Fae		E-Fae	
	Relative activity (%)	Specific activity (µM/min/µg)	Relative activity (%)	Specific activity (µM/min/µg)	Relative activity (%)	Specific activity (µM/min/µg)
Methyl ferulate	70.0 ± 7.3	7.5 ± 0.8	74.4 ± 3.9	3.8 ± 0.2	59.6 ± 19.8	1.3 ± 0.4
Methyl caffeate	85.3 ± 23.4	9.1 ± 2.5	24.2 ± 2.0	1.2 ± 0.1	Trace^a^	Trace^a^
Methyl sinapate	44.2 ± 6.4	4.7 ± 0.7	19.8 ± 5.3	1.0 ± 0.3	28.3 ± 15.4	0.6 ± 0.3
Methyl *p*-coumarate	100 ± 20.0	10.7 ± 2.1	100 ± 7.7	5.1 ± 0.4	100 ± 10.5	2.1 ± 0.2

Data were obtained at 35 °C in sodium phosphate at pH 7.5 for M-Fae and E-Fae, and at pH 8.0 for P-Fae, using a stopped assay. The results given are the average of three experiments ± one standard deviation

^a^Trace: trace activity observed (less than 0.4 µM/min/µg)

Activity profiles were determined as a function of reaction temperature and reaction pH (Fig. 4b and Additional file 1: Figure S7). No significant differences among the immobilized enzymes versions, nor compared to their free counterparts, were observed when varying the reaction pH (Additional file 1: Figures S5, S7). When varying the reaction temperature, the immobilized M-Fae showed the highest value of T_opt_ (55 °C), and unlike the other two versions, M-Fae value of T_opt_ was unchanged when immobilized (Fig. 4). The T_opt_ value for immobilized P-Fae remained higher than that of immobilized E-Fae (25 °C and 20 °C, respectively, Fig. 4b), but was much lower than that of free P-Fae (45 °C) (Fig. 4a).

## Discussion

Glycan synthesis pathways, and therefore enzymes glycan structures are known to differ between organisms (Nadeem et al. 2018). In this study we investigated the differences of glycosylation patterns of an enzyme produced in two host organisms, *M. thermophila* and *P. pastoris*. Glycan analysis by MS showed that, on average, P-Fae *N*-glycans were larger than those of M-Fae and confirmed that both *N*-glycosylation sites carried glycans. The longer *N*-glycan chains observed on *Mt*Fae1a when it was produced in *P. pastoris* suggest that this yeast may possess a different cell machinery; in particular the involvement of different glycosyltransferases and glycosidases in glycan processing (Gupta and Shukla 2018).

Protein *N*-glycosylation is known to affect protein folding (Benoit et al. 2006) and thermal stability (Chu et al. 1978). The presence of glycan chains on a protein surface has also been suggested to reduce the exposure of hydrophobic residues, which could, in turn, lead to reduced protein aggregation, as observed for a *Saccharomyces cerevisiae* invertase (Schülke and Schmid 1988). In the present study, the non-glycosylated version, produced in *E. coli* (E-Fae), was mostly found in insoluble aggregates despite co-expression with chaperones (data not shown).

*N*-Glycosylation has been shown to modify the folding process (through modulation of folding intermediates), and may influence the folded state conformational populations (Hoffmann and Flörke 1998; Hanson et al. 2009; Skropeta 2009). *N*-Glycosylation may also influence catalysis by slowing down the overall protein-structure dynamics (Rudd et al. 1994; Lee et al. 2015). The specific activities of the two glycosylated enzymes, M-Fae and P-Fae, were up to six times higher than that of the non-glycosylated E-Fae, on all substrates. The observed variations in specificity and activity could be due to differences in glycosylation. The location of the glycosylation site might also explain activity differences, since *N*-glycosylation close to the active site of some proteases has also been shown to alter their substrate binding and turnover (Goettig 2016). A similar phenomenon may be occurring with glycosylation of Asn179, located less than 15 Å away from the three catalytic residues in *Mt*Fae1a.

Glycosylation was found to have a major effect on the thermal stability of the *Mt*Fae1a versions. The natively glycosylated M-Fae displayed the highest T_m_ and T_opt_ values (60 and 55 °C, respectively), whereas the non-glycosylated E-Fae was the least tolerant to high temperatures, showing the lowest values of T_m_ and T_opt_. Glycosylation has been reported to have positive effects on the thermal stability of numerous proteins, and some underlying mechanisms have been proposed. It has been suggested that stabilization could occur through glycan–protein contacts between the *N*-linked core triose and surrounding amino acids (Hanson et al. 2009), or that *N*-glycans could stabilize proteins through slower protein-structure dynamics (Lee et al. 2015). *N*-Glycosylation has also been shown to facilitate oligomerization (Mitra et al. 2006), which could play an important role in structure stability since *Mt*Fae1a was predicted to form a homo-dimer and to have one glycosylation site located at the dimerization interface (Asn117).

Protein immobilization by physical adsorption relies on surface properties of enzyme and support, mostly through electrostatic interactions. Therefore, pH at which immobilization is performed can greatly affect the immobilization kinetics (Thörn et al. 2013). The theoretical isoelectric point (pI) of the support material, has been determined to be 3.8 (Hudson et al. 2005), which implies that the overall charge on the support was negative under all the conditions tested. The pI of *Mt*Fae1a was theoretically determined to be 4.9, while that of M-Fae has been experimentally determined to be 6.0 (Kühnel et al. 2012). M-Fae followed the expected pH-dependent immobilization behavior. P-Fae also exhibited pH-dependent immobilization, but could be immobilized at higher pH values than M-Fae. This behavior could be due to a higher pI for P-Fae (which was not experimentally determined), but might also be due to alteration of the apparent pI through shielding of protein surface by glycan-chains (Li et al. 2017). Shielding of the surface charges might also reduce the repulsion between proteins, since P-Fae was shown to harbor longer glycan chains than M-Fae, this could explain the faster immobilization of P-Fae. The immobilization of E-Fae was not pH-dependent, since immobilization was almost immediate at all pHs tested. E-Fae had the smallest apparent size due to its lack of glycosylation, and probably diffused more easily into the 10 nm pores of the mesoporous material. Glycosylation of M-Fae and P-Fae increased their apparent molecular weight, and probably also their hydrodynamic volume (Woods 2018).

Immobilization has been suggested to induce some conformational changes on the structure of enzymes (Hlady and Buijs 1996), which can lead to decreased enzymatic activity. M-Fae was the least affected by immobilization in terms of specific activity and its T_opt_ value was not affected. These findings suggest that the native glycosylation pattern of M-Fae may protect the enzyme structure from immobilization adverse effects. The behavior of M-Fae, compared to the other *Mt*Fae1a versions, strongly suggests that the composition of the glycan chains carried by the enzyme affected its behavior. Thermostability of enzymes is an important aspect for their industrial applicability. Interestingly, M-Fae displayed a 10 °C higher T_opt_ value, and an 8 °C higher T_m_ value than the enzyme version recombinantly produced in *P. pastoris*, P-Fae. The difference was even greater when the enzymes were immobilized, with M-Fae displaying a 30 °C higher T_opt_, than P-Fae. Careful production host selection is consequently a tool that can help in producing thermostable enzymes, and could alleviate or remove the need for enzyme engineering for thermostability. Strain engineering strategies could also be used to enable the heterologous hosts to produce glycan chains more similar to those of the native host. This approach, although tedious, has been used in pharmacological applications, where the production of human-like glycans has been achieved in *P. pastoris* and *E. coli* (Gerngross 2004; Wayman et al. 2019).

In conclusion, we characterized the differences in the glycan chains of enzymes produced by two microorganisms, *M. thermophila* and *P. pastoris*. Glycosylation levels and patterns explained most of the differences observed among the enzymes versions, and our findings demonstrated that the length and composition of the glycan chains led to differences in behavior between M-Fae and P-Fae. We thus demonstrated the importance of comparing production hosts and examining the properties of the resulting enzymes, especially when using them in high-temperature processes, such as enzymatic hydrolysis of biomass.

## Additional file

**Additional file 1. Figure S1 ** Examples of MS/MS spectra for an *N*-glycopeptide **Table S1.** Relative glycoform distribution for Asn179 (NQT). **Table S2**. Relative glycoform distribution for Asn117 (NYT) Figure S2 Enzyme kinetics for the three *Mt*Fae1a versions. **Figure S3**. Data points and fitting curves used during non-linear regression for calculations of the melting temperature. **Figure S4**. Visualization of the amino acids forming the catalytic triad and of the two glycosylated asparagine residues on a homology model. **Table S3**. Estimated relative distribution (%) of glycosylation site occupancy for the two glycosylated *Mt*Fae1a preparations Figure S5 pH-dependent activity profiles of the three *Mt*Fae1a versions. **Figure S6**. Residual activities of the three *Mt*Fae1a versions incubated at various pHs. **Table S4**. Specific activities of the three immobilized *Mt*Fae1a versions depending on the immobilization pH. **Figure S7**. pH-dependent activity profiles of the immobilized *Mt*Fae1a versions.

## Data Availability

All data generated or analyzed during this study are included in this published article and its additional file or available from the corresponding author on reasonable request.

## Abbreviations

CV: column volume
DMSO: dimethyl sulfoxide
EIC: extracted ion chromatograph
FAE: feruloyl esterase
His_6_-tag: hexa histidine tag
IEX: ion exchange chromatography
LC: liquid chromatography
LB: lysogeny broth
MCA: methyl caffeate
MFA: methyl ferulate
M*p*CA: methyl *p*-coumarate
MPS: mesoporous silica
MSA: methyl sinapate
MS: mass spectrometry
MW: molecular weight
OD_600_: optical density at 600 nm
*p*CA: *p*-coumaric acid
SA: specific activity
SBA-15: Santa Barbara Amorphous type 15
SDS-PAGE: sodium dodecyl sulfate polyacrylamide gel electrophoresis
TEAB: triethylammonium bicarbonate
