# Supplementary material for: Glycosylation influences activity, stability and immobilization of the feruloyl esterase 1a from Myceliophthora thermophila
Source: AMB Express. 2019 Aug 12;9:126. doi: 10.1186/s13568-019-0852-z (PMC6691016; doi:10.1186/s13568-019-0852-z)
Supplement: Supplementary file 1 — Additional file 1. Figure S1 Examples of MS/MS spectra for an N-glycopeptide Table S1. Relative glycoform distribution for Asn179 (NQT). Table S2. Relative glycoform distribution for Asn117 (NYT) Figure S2 Enzyme kinetics for the three MtFae1a versions. Figure S3. Data points and fitting curves used during non-linear regression for calculations of the melting temperature. Figure S4. Visualization of the amino acids forming the catalytic triad and of the two glycosylated asparagine residues on a homology model. Table S3. Estimated relative distribution (%) of glycosylation site occupancy for the two glycosylated MtFae1a preparations Figure S5 pH-dependent activity profiles of the three MtFae1a versions. Figure S6. Residual activities of the three MtFae1a versions incubated at various pHs. Table S4. Specific activities of the three immobilized MtFae1a versions depending on the immobilization pH. Figure S7. pH-dependent activity profiles of the immobilized MtFae1a versions. [file 13568_2019_852_MOESM1_ESM.pdf]

Additional file 1.

AMB express

Glycosylation influences activity, stability and immobilization of the feruloyl esterase 1a from *Myceliophthora thermophila*

Cyrielle Bonzom[1], Silvia Hüttner[1], Ekaterina Mirgorodskaya[2], Sun-Li Chong[1#], Stefan Uthoff[3], Alexander Steinbüchel[3,4], Raymond M.D. Verhaert[5], Lisbeth Olsson[1]*

From the [1]Division of Industrial Biotechnology, Department of Biology and Biological Engineering, Chalmers University of Technology, SE-412 96 Gothenburg, Sweden, [2]Proteomics Core Facility, Sahlgrenska Academy, University of Gothenburg, SE-405 30 Gothenburg, Sweden, [3]Institut für Molekulare Mikrobiologie und Biotechnologie, Westfälische Wilhelms-Universität Münster, Corrensstraβe 3, DE-48149 Münster, Germany, [4]Department of Environmental Sciences, King Abdulaziz University, Jeddah, Saudi Arabia, [5]ProteoNic BV, J.H. Oortweg 19-21, NL-2333 CH Leiden, the Netherlands

[#]**Present address:** State Key Laboratory of Subtropical Silviculture, School of Forestry and Biotechnology, Zhejiang Agriculture and Forestry University, Linan, 311300, P.R. China

***To whom correspondence should be addressed:** Lisbeth Olsson: Division of Industrial Biotechnology, Department of Biology and Biological Engineering, Chalmers University of Technology, SE-412 96 Gothenburg, Sweden; lisbeth.olsson@chalmers.se; Tel. +46(0) 31 772 3805; Fax. +46(0) 31 772 3801.

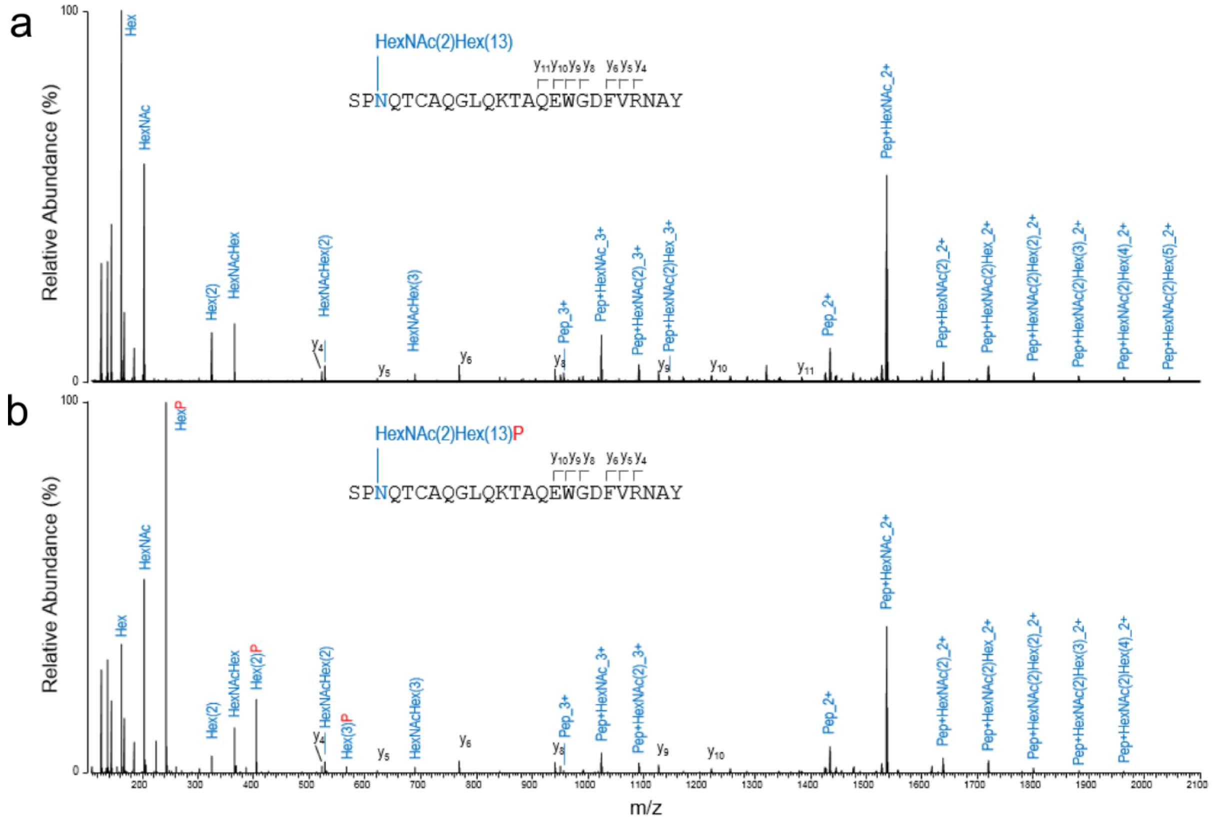

Figure S1 Examples of MS/MS spectra for an *N*-glycopeptide Glycopeptide SPNQTCAQGLQKTAQEWGDFVRNAY, **(a)** from M-Fae, carrying a non-phosphorylated high-mannose glycan: HexNAc(2)Hex(13), **(b)** from P-Fae, carrying a phosphorylated high-mannose glycan: HexNAc(2)Hex(13)P. Peptide identification was confirmed by the detected series of y-fragment ions along the peptide chain. Hex: hexose.

Table S1 Relative glycoform distribution for Asn179 (NQT)

Glycan structure	M-Fae	P-Fae
$HexNAc_2Hex_4$	0.06 ± 0.10	NO
$HexNAc_2Hex_5$	0.56 ± 0.52	NO
$HexNAc_2Hex_6$	0.28 ± 0.26	NO
$HexNAc_2Hex_7$	1.97 ± 0.38	0.01 ± 0.02
$HexNAc_2Hex_8$	34.77 ± 5.52	10.32 ± 2.07
$HexNAc_2Hex_9$	3.60 ± 1.68	56.57 ± 4.02
$HexNAc_2Hex_{10}$	56.67 ± 7.23	15.83 ± 1.14
$HexNAc_2Hex_{11}$	2.09 ± 0.74	7.95 ± 0.78
$HexNAc_2Hex_{12}$	NO	7.09 ± 1.04
$HexNAc_2Hex_{13}$	NO	1.51 ± 0.41
$HexNAc_2Hex_{14}$	NO	0.43 ± 0.40
$HexNAc_2Hex_{15}$	NO	0.29 ± 0.22

Values are given as a percentage of all observed glycopeptides containing the Asn179 (NQT) glycosylation site. Results are presented as averages ± one standard deviation. NO: Not Observed. Hex: hexose. Due to differences in ionization efficiency and retention time between high-mannose- and mannose-phosphate-containing glycopeptides, mannose-phosphate-containing glycopeptides were excluded from the calculation of the glycoform distribution. The relative glycoform abundances were calculated as an average of the observed intensity for three chymotriptic peptides: "SP**NQT**CAQGLQKTAQEWGDFVRNAY", "SP**NQT**CAQGLQKTAQEWGDFVRNAYAGY", and "GCAAGAESATPFSP**NQT**CAQGLQKTAQEWGDFVRNAY".

Table S2 Relative glycoform distribution for Asn117 (NYT)

Glycan structure	M-Fae	P-Fae
$HexNAc_2Hex_1$	0.09 ± 0.12	NO
$HexNAc_2Hex_2$	0.07 ± 0.11	NO
$HexNAc_2Hex_3$	5.32 ± 7.35	NO
$HexNAc_2Hex_4$	23.99 ± 23.74	0.12 ± 0.16
$HexNAc_2Hex_5$	44.24 ± 32.20	NO
$HexNAc_2Hex_6$	11.11 ± 4.53	NO
$HexNAc_2Hex_7$	8.14 ± 0.51	NO
$HexNAc_2Hex_8$	4.30 ± 1.09	0.18 ± 0.26
$HexNAc_2Hex_9$	2.11 ± 2.23	6.86 ± 2.96
$HexNAc_2Hex_{10}$	0.44 ± 0.54	13.07 ± 2.97
$HexNAc_2Hex_{11}$	0.20 ± 0.29	14.04 ± 0.77
$HexNAc_2Hex_{12}$	NO	13.13 ± 0.19
$HexNAc_2Hex_{13}$	NO	11.73 ± 1.67
$HexNAc_2Hex_{14}$	NO	8.11 ± 1.57
$HexNAc_2Hex_{15}$	NO	11.11 ± 0.9
$HexNAc_2Hex_{16}$	NO	5.46 ± 0.02
$HexNAc_2Hex_{17}$	NO	2.2 ± 1.07
$HexNAc_2Hex_{18}$	NO	2.69 ± 1.81
$HexNAc_2Hex_{19}$	NO	1.98 ± 0.5
$HexNAc_2Hex_{20}$	NO	1.9 ± 1.28
$HexNAc_2Hex_{21}$	NO	1.35 ± 1.46
$HexNAc_2Hex_{22}$	NO	0.29 ± 0.41

Values are given as a percentage of all observed glycopeptides containing the Asn117 (NYT) glycosylation site. Results are presented as averages ± one standard deviation. NO: Not Observed. Hex: hexose. Due to differences in ionization efficiency and retention time between high-mannose- and mannose-phosphate-containing glycopeptides, mannose-phosphate-containing glycopeptides were excluded from the calculation of the glycoform distribution. The relative glycoform abundances were calculated as an average of the observed intensity for two chymotriptic peptides: "DIQNPDTLTHGQGGDALGIVSMV**NYT**LDKHSGDSSRVY", "DIQNPDTLTHGQGGDALGIVSMV**NY**". The chymotrypsin cleavage efficiency seemed to be strongly impaired by the presence of large high-mannose structures close to its cleaving site. This could explain some of the large standard deviations observed.

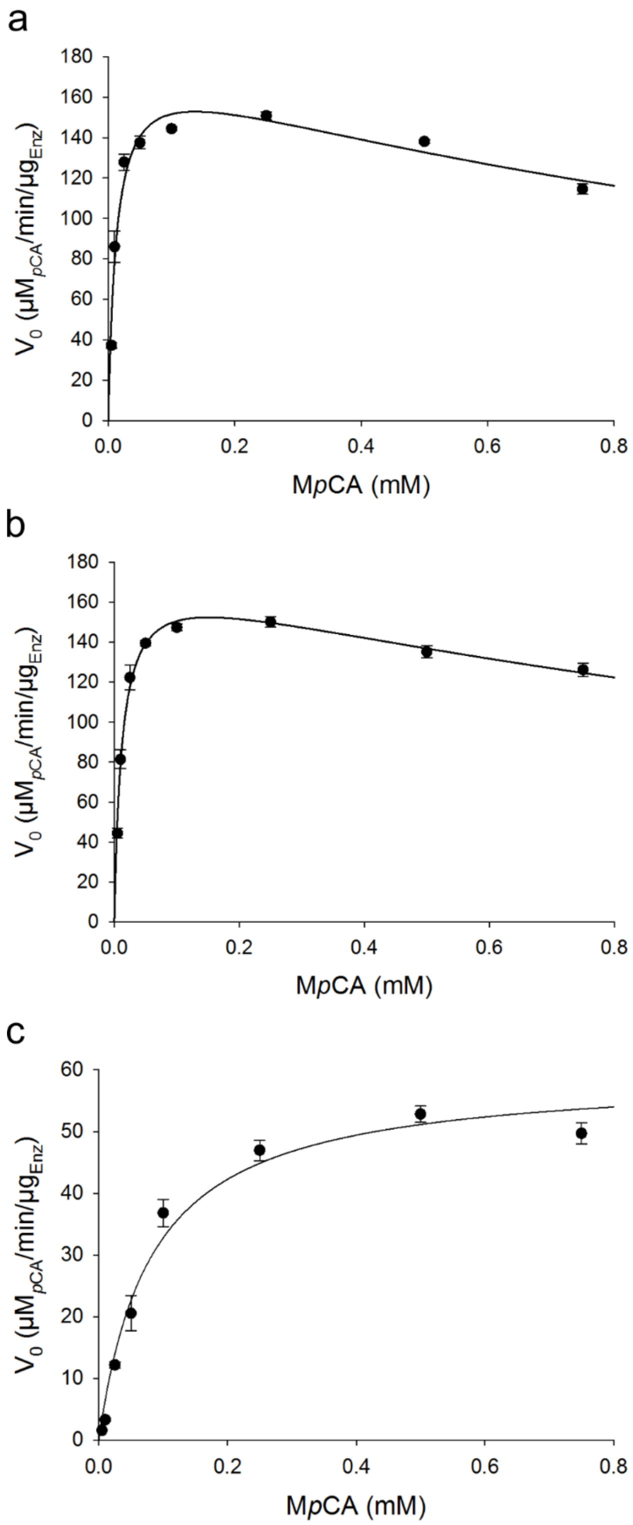

Figure S2 Enzyme kinetics for the three *Mt*Fae1a versions (a) M-Fae, **(b)** P-Fae, **(c)** E-Fae. V_0 (initial velocities) was plotted depending on the initial substrate concentration (M*p*CA). Activities were measured at 35°C in a continuous assay. The data were first fitted to the Michaelis-Menten equation, Equation (1), allowing the determination of K_m and V_{max}. If the data did not fit Equation (1), substrate inhibition was taken into account. This was done by fitting the data to the substrate inhibition equation (Equation (2)) which returned the parameter K_{si} in addition to K_m, and V_{max}. k_{cat} was then calculated by dividing V_{max} by the initial molar concentration of the enzyme.

$$V = \frac{V_{max}[S]}{K_m+[S]} \qquad (1)$$

$$V = \frac{V_{max}\,[S]}{K_m+[S](\frac{1+[S]}{K_{si}})} \qquad (2)$$

V is the measured initial reaction rate and [S] is the substrate concentration. **(a)** and **(b)** were fitted using the Michaelis-Menten substrate inhibition equation (Equation (2)), **(c)** was fitted using the Michaelis-Menten equation (Equation (1)). Results are presented as the mean values of triplicate experiments, and error bars represent one standard deviation.

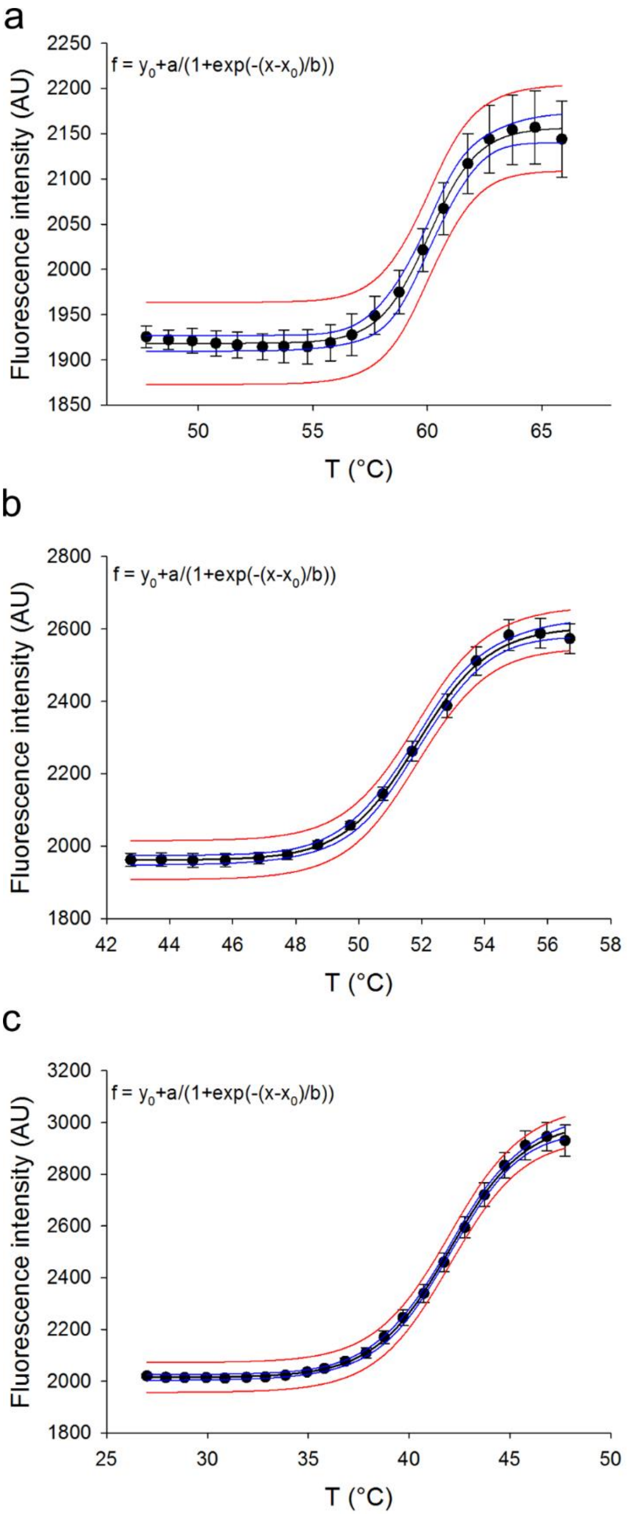

Figure S3 Data points and fitting curves used during non-linear regression for calculations of the melting temperature (a) M-Fae. **(b)** P-Fae. **(c)** E-Fae. Black: data points, blue: 95% confidence band, red: 95% prediction band. AU: Arbitrary Units. The data, from the relevant part of the curve, were fitted by non-linear regression to a 4-parameter sigmoid curve (Equation (3)) using SigmaPlot software.

$$y = y_0 + \frac{a}{1+\exp\frac{-(x-x_0)}{b}} \quad (3)$$

Where, y_0 is the minimum fluorescence intensity, a is the difference between the maximum and minimum fluorescence intensities measured, b is the slope of the curve around T_m, and $x_0 = T_m$. Results are presented as the mean values of triplicate experiments, and error bars represent one standard deviation.

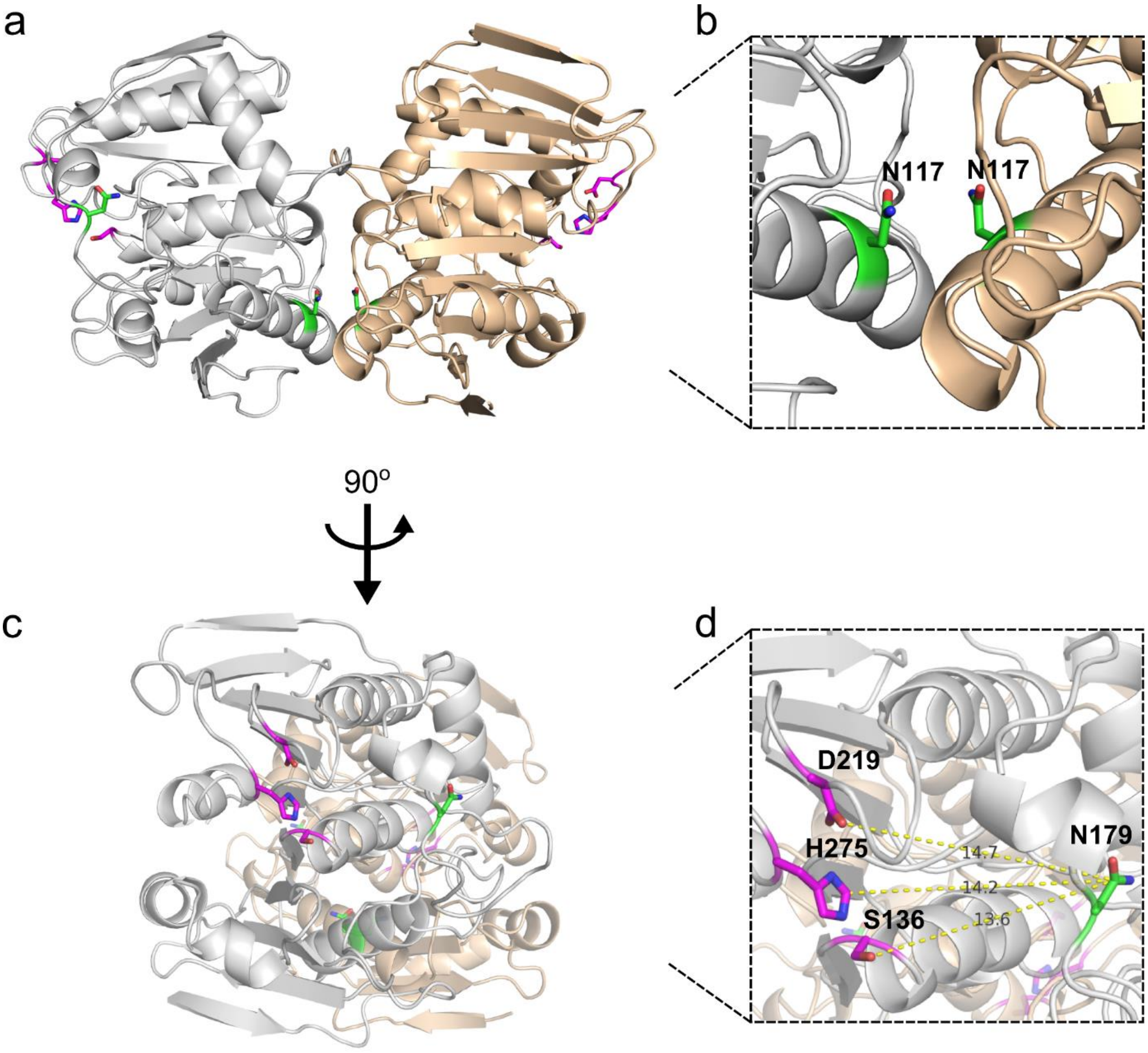

Figure S4 Visualization of the amino acids forming the catalytic triad and of the two glycosylated asparagine residues on a homology model (a) The overall structure showing the proposed dimer biological unit (grey and copper colors). **(b)** The location of glycosylated Asn117 (green) is predicted to be at the dimer interface and symmetry axis. **(c)** Rotation of the overall structure (90° with respect to a**)**. **(d)** The glycosylated Asn179 (green) is predicted to be close to the catalytic residues Ser136, His275 and Asp219 (magenta), dotted lines represent distance (in Angstroms) between residues. The homology model was deposited by Topakas *et al.* (Topakas et al. 2012) and recovered from the Swiss Model repository (SMR ID: G2QND5). Visualization and images were obtained using the PyMol software (Schrödinger).

Table S3 Estimated relative distribution (%) of glycosylation site occupancy for the two glycosylated *Mt*Fae1a preparations

	Asn179 (NQT)			Asn117 (NYT)		
	NG	High-man	man-P	NG	High-man	man-P
M-Fae	NO	100	NO	0.2 ± 0.2	99.8 ± 0.2	NO
P-Fae	NO	98.9 ± 0.7	1.5 ± 0.7	3.3 ± 4.7	90.2 ± 6.1	6.5 ± 1.4

Values given are the percentage of all observed peptides/glycopeptides carrying the potential glycosylation site. Results are averages ± one standard deviation. NG: Non-Glycosylated, High-man: high-mannose structure, man-P: mannose-phosphate containing structures, NO: Not Observed. Due to different ionization efficiency and different retention times among non-glycosylated as well as high-man- and man-P-containing glycopeptides, only estimates of the relative distribution between these groups were possible for each site.

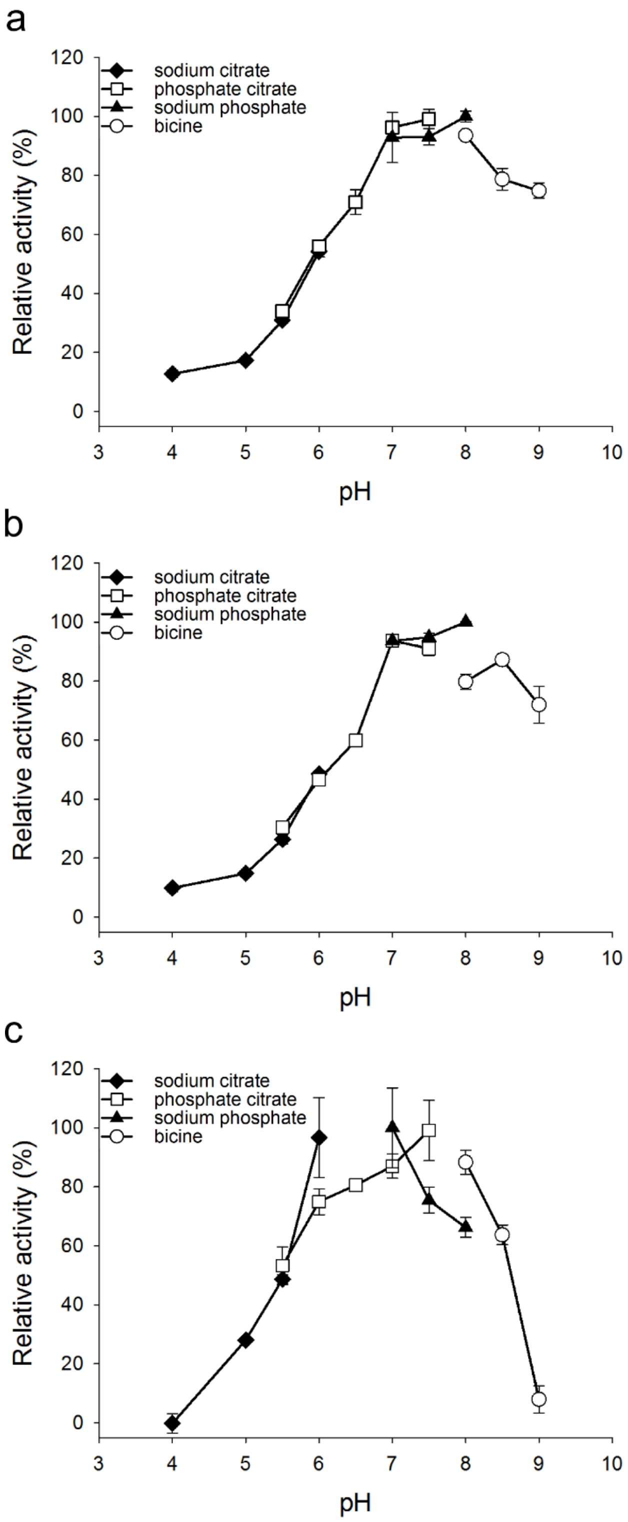

Figure S5 pH-dependent activity profiles of the three *Mt*Fae1a versions (a) M-Fae. **(b)** P-Fae. **(c)** E-Fae. pH profiles were determined by measuring the activity at 30°C, using the standard continuous assay. The activities obtained are presented as a percentage of the highest activity observed. Results are averages of three experiments, and error bars represent one standard deviation.

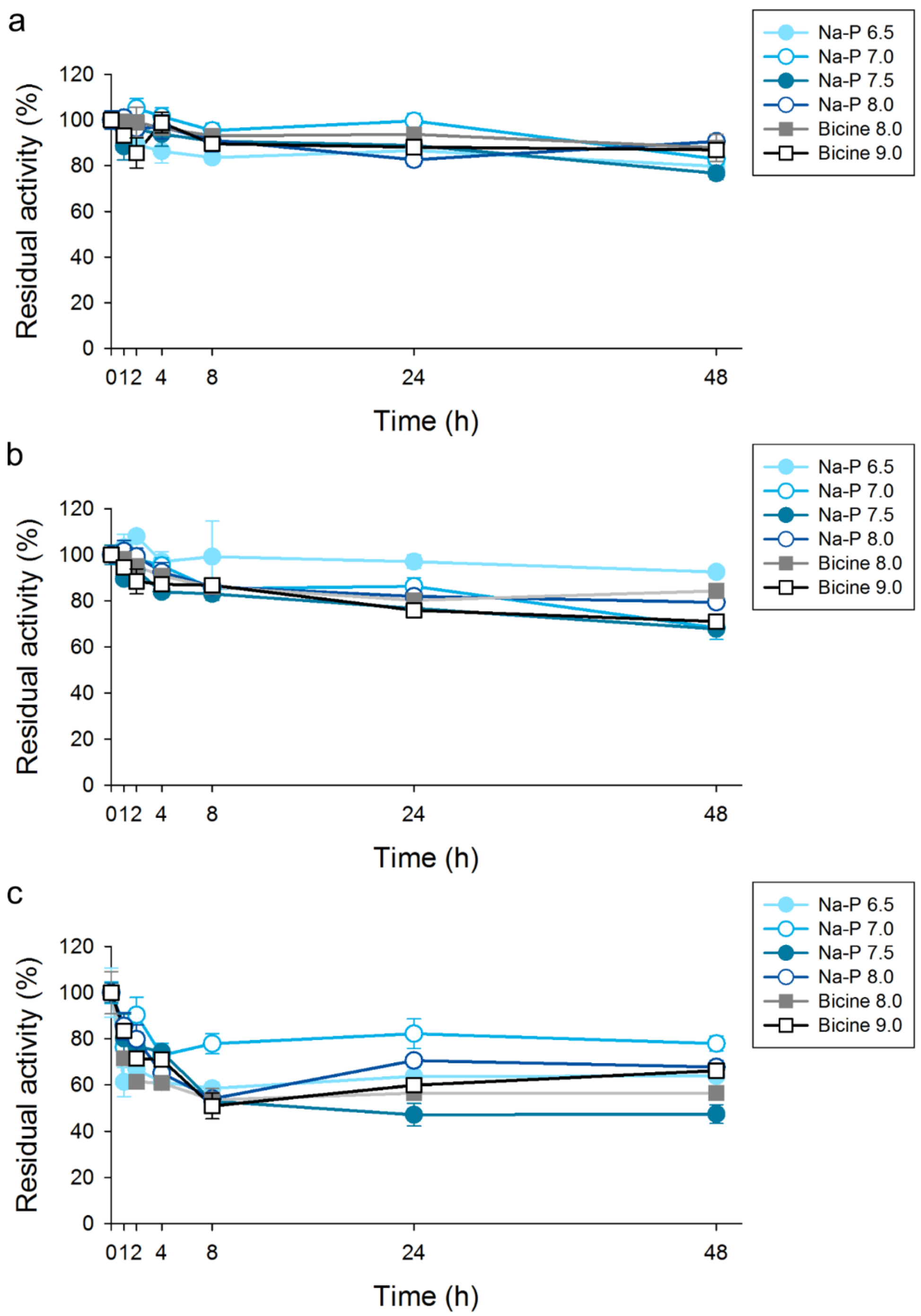

Figure S6 Residual activities of the three *Mt*Fae1a versions incubated at various pHs (a) M-Fae. **(b)** P-Fae. **(c)** E-Fae. The three enzyme versions were incubated in 100 mM sodium phosphate (Na-P) and bicine buffer at 20°C for 48 h. The activities measured at each time point are expressed as the percentage of activity remaining relative to the initial activity. Results are presented as the mean values of triplicate experiments, and error bars represent one standard deviation.

Table S4 Specific activities of the three immobilized *Mt*Fae1a versions depending on the immobilization pH

Enzyme version	Immobilization pH	Specific activity (µM/min/µg)
M-Fae	5.0	5.5 ± 0.5
	6.0	8.4 ± 1.5
	7.0	0.5 ± 0.4
P-Fae	5.0	0.8 ± 0.1
	6.0	3.6 ± 0.3
	7.0	0.3 ± 0.01
E-Fae	5.0	0.1 ± 0.1
	6.0	1.2 ± 0.1
	7.0	0.8 ± 0.6

Immobilization was performed in phosphate citrate at 20°C for 24 h. Activity data were obtained in sodium phosphate buffer (pH 7.0) at 35°C, using the standard stopped assay. Results given are the average of three experiments ± one standard deviation.

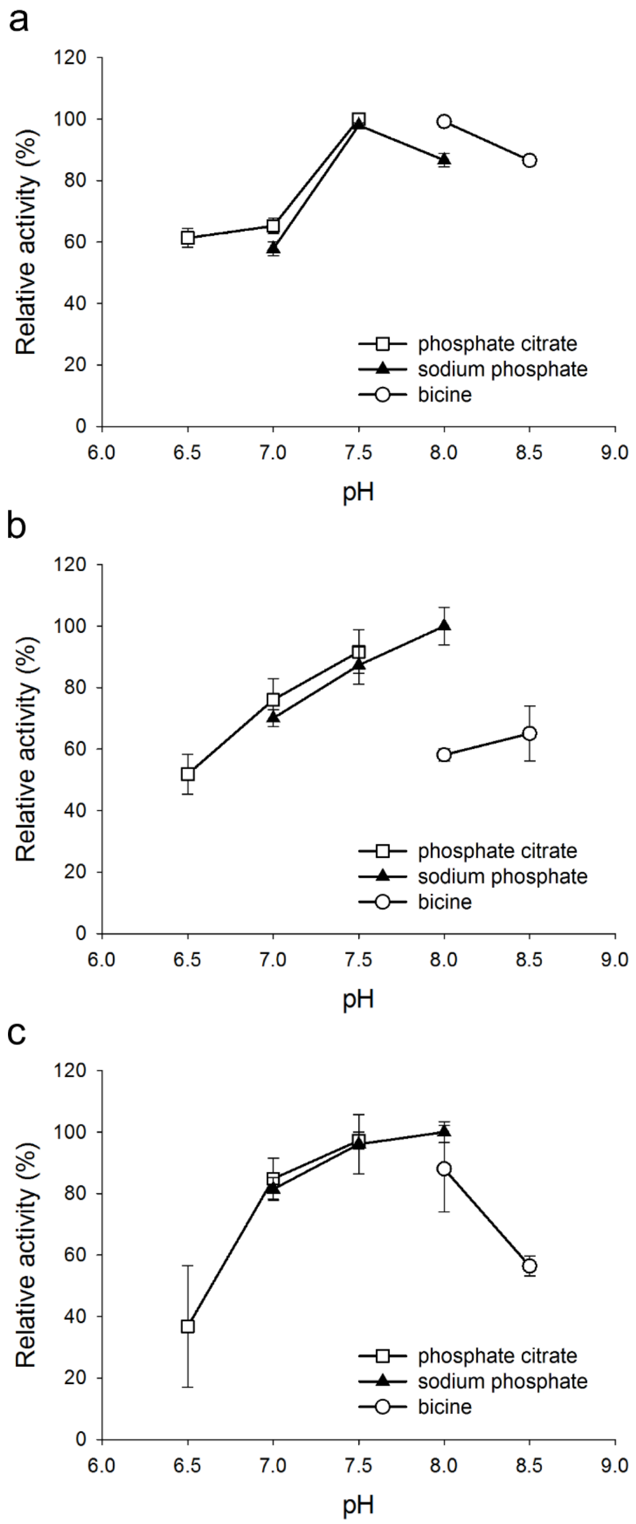

Figure S7 pH-dependent activity profiles of the immobilized *Mt*Fae1a versions (a) M-Fae. **(b)** P-Fae. **(c)** E-Fae. Phosphate citrate buffer at pH 6.5-7.5 (open squares), sodium phosphate buffer at pH 7.0-8.0 (filled triangles), and bicine buffer at pH 8.0-8.5 (open circles). pH profiles were determined after immobilization in sodium phosphate buffer at pH 6.0 by measuring the activity at 35°C, using the standard stopped assay. The activities obtained are presented as a percentage of the highest activity observed. Results are the average of triplicate experiments, and error bars represent one standard deviation.

References

Topakas E, Moukouli M, Dimarogona M, Christakopoulos P (2012) Expression, characterization and structural modelling of a feruloyl esterase from the thermophilic fungus *Myceliophthora thermophila*. Appl Microbiol Biotechnol 94:399–411 . doi: 10.1007/s00253-011-3612-9
